# Erratum for "Polypoid Cystitis: A Retrospective Case‐Series of 112 Dogs”

**DOI:** 10.1111/jvim.70155

**Published:** 2025-06-18

Price MP , Thomas R , Breen M , Kendall AR , Vaden SL , Polypoid Cystitis: A Retrospective Case‐Series of 112 Dogs. J Vet Intern Med. 2025 May‐Jun;39(3):e70049, doi: 10.1111/jvim.70049 PMC1208182140375558 PMID: 40375558; PMCID: PMC12081821.

In the above‐mentioned article, Table 1 “Key characteristics of the study cohort” has an incorrect layout. There are three sections to the table as shown here.

**TABLE 1:** Key characteristics of the study cohort.

Sex	Male castrated:	Male Intact	Female Spayed	Female Intact
	n= 34	n= 6	n= 64	n=8
**Polyp Location**	**Cranioventral**	**Trigone**	**Multifocal**	**Location not described**
	n= 56	n=9	n=1	n=4
**Comorbidity**	**UTI**	**Urocystolithiasis**	**UTI + Urocystolithiasis**	**Unknown**
	n= 61	n=38	n=14	n=16

We apologize for this error.

